# Differential Evolutionary History in Visual and Olfactory Floral Cues of the Bee-Pollinated Genus *Campanula* (Campanulaceae)

**DOI:** 10.3390/plants10071356

**Published:** 2021-07-02

**Authors:** Paulo Milet-Pinheiro, Pablo Sandro Carvalho Santos, Samuel Prieto-Benítez, Manfred Ayasse, Stefan Dötterl

**Affiliations:** 1 Institute of Evolutionary Ecology and Conservation Genomics, University of Ulm, Albert-Einstein Allee, 89081 Ulm, Germany; pablo.santos@uni-ulm.de (P.S.C.S.); manfred.ayasse@uni-ulm.de (M.A.); 2 Departamento de Biología y Geología, Física y Química Inorgánica, Universidad Rey Juan Carlos-ESCET, C/Tulipán, s/n, Móstoles, 28933 Madrid, Spain; samuel.prieto@urjc.es; 3 Ecotoxicology of Air Pollution Group, Environmental Department, CIEMAT, Avda. Complutense, 40, 28040 Madrid, Spain; 4 Department of Biosciences, Paris-Lodron-University of Salzburg, Hellbrunnerstrasse 34, 5020 Salzburg, Austria; stefan.doetterl@sbg.ac.at

**Keywords:** bee olfaction, bee vision, *Campanula*, floral trait evolution, floral color, floral scents, mutualism, phylogenetic PCA, phylogenetic inertia, pollinator-mediated selection

## Abstract

Visual and olfactory floral signals play key roles in plant-pollinator interactions. In recent decades, studies investigating the evolution of either of these signals have increased considerably. However, there are large gaps in our understanding of whether or not these two cue modalities evolve in a concerted manner. Here, we characterized the visual (i.e., color) and olfactory (scent) floral cues in bee-pollinated *Campanula* species by spectrophotometric and chemical methods, respectively, with the aim of tracing their evolutionary paths. We found a species-specific pattern in color reflectance and scent chemistry. Multivariate phylogenetic statistics revealed no influence of phylogeny on floral color and scent bouquet. However, univariate phylogenetic statistics revealed a phylogenetic signal in some of the constituents of the scent bouquet. Our results suggest unequal evolutionary pathways of visual and olfactory floral cues in the genus *Campanula*. While the lack of phylogenetic signal on both color and scent bouquet points to external agents (e.g., pollinators, herbivores) as evolutionary drivers, the presence of phylogenetic signal in at least some floral scent constituents point to an influence of phylogeny on trait evolution. We discuss why external agents and phylogeny differently shape the evolutionary paths in floral color and scent of closely related angiosperms.

## 1. Introduction

Pollination is a critical step for successful sexual reproduction in flowering plants [1]. It is sometimes performed by abiotic agents, such as wind and water [2,3], but mostly by biotic agents, i.e., animals [4]. Angiosperms typically offer floral resources (mainly pollen and nectar) as a reward for pollination services [1]. Flowers advertise these rewards through floral signals, such as color and scent, which, as a consequence, are the subjects of selective pressures by their pollinators [5,6,7]. Pollinator-mediated selection has led to the convergent evolution of similar floral traits (color and scent) in unrelated angiosperm species that share the same guild of pollinators (pollination syndromes; sensu [8]).

Melittophily, i.e., pollination by bees, is the most widespread and variable pollination syndrome among flowering plants. Bees are an unusually diverse group of pollinators with about 20,000 species [9], differing strongly not only in aspects such as size, shape, and social behavior, but also in their sensory capabilities [10,11]. Accordingly, floral traits of bee-pollinated flowers vary considerably, making the delineation of a single signature of this syndrome somewhat inappropriate [12]. In spite of this great variability, some traits are more easily attributed to bee-pollinated flowers than others [13], suggesting that distinct floral traits experience unequal selective pressures by pollinating bees. Flower color, for example, is predominantly blue and yellow and is generally a good predictor of bee-pollinated flowers, especially when also considering morphological traits [8]. In contrast, evidence is lacking for similarly converging patterns on floral scent chemistry in this pollination syndrome [14].

Bees generally have a trichromatic color vision, with photoreceptors sensitive to ultra-violet (UV), blue, and green wavelengths [15]. The visual system of bees is highly conserved [15,16] and predates the evolution of angiosperms [17]. Compared with color vision, the bee’s olfactory system is substantially more complex and variable at the receptor level. Bees have many more olfactory than color receptors and thus potentially have a remarkable olfactory ability to perceive a broad range of scent compounds and blends thereof [18]. There is variation in the number of receptors among species; for example, the pollen-generalist Western honeybee, *Apis mellifera*, has about 160 olfactory receptors [18], whereas the pollen-specialist bee *Andrena vaga* has about 130 [19]. The striking difference concerning the complexity of visual vs. olfactory sensory ability suggests that color and scent in bee-pollinated flowers have undergone different selective pressures, and this might be particularly true for plants that rely on several bee species as pollinators. In other words, whereas the selection of color might have been strongly constrained by the conserved visual system of bees, the evolution of floral scents might have been less constrained, reflecting the great variability in the olfactory system of different bee species. So far, however, little is known on whether visual and olfactory cues evolved in a concerted manner in closely related mellitophilous plants [20].

The mellitophilous genus *Campanula* L. (Campanulaceae), popularly known as bellflowers, bluebells, harebells and starbells, is well suited to assess the evolutionary history of visual and olfactory cues in a comparative manner. It comprises ca. 400 species worldwide [21] that offer nectar and pollen to their bee pollinators [22,23,24]. Flowers of *Campanula* are generally attractive to a broad spectrum of bees (e.g., *Andrena* spp., *Bombus* spp., *Chelostoma* spp., *Lassioglossum* spp., *Osmia* spp.) and, in a given community, the different *Campanula* species usually attract the same bees. The differences observed in pollinating fauna are generally attributable to a variation in the relative abundance of the different bee species rather than to their presence/absence [23]. Flowers of *Campanula* are classified as rotate or subrotate, broadly campanulate or tubular-campanulate; such morphology might restrict the access of possible pollinators to resources [24]. Nevertheless, even in the more specialized tubular-campanulate flowers, flower visits by bees belonging to four to five families have frequently been reported [23,24,25]. For a long time, the evolution of floral traits was assumed to be shaped by the most effective pollinator principle [26]. A few decades later, however, Aigner [27] proposed other evolutionary models in which phenotypic selection is not only mediated by the most effective pollinators but is rather a result of the additive effects of all floral visitors within pollinator communities. In *Campanula*, therefore, the evolution of floral traits is likely to experience distinct selective pressures exerted simultaneously by flower-visiting bees from several different taxa that vary immensely in their morphological, behavioral, and sensory traits [10,28,29,30,31].

In the present study, we have integrated phylogenetic, spectrophotometric, and chemical analyses in order to investigate signatures of evolution of floral color and scent in 14 and 11 species of *Campanula*, respectively. Since co-occurring *Campanula* species usually have a diverse and similar assemblage of flower-visiting bees (although in different proportions of species; see above) and knowing that bees of different taxa (genera/families) differ considerably with respect to olfaction [19,32], but not to vision [15,17], we expected that floral scent and color have undergone differential evolutionary histories. Taking this perspective, we have investigated whether both signal modalities are similarly or differently influenced by phylogenetic constraints.

## 2. Results

### 2.1. Comparisons of Floral Colour Reflectance among Campanula Species

**Figure 1:**
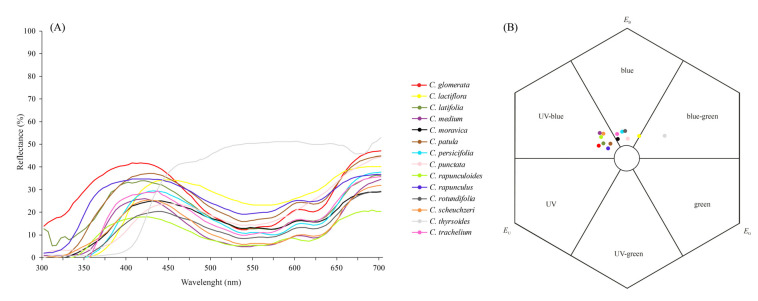
Mean spectral reflectance curves of 14 *Campanula* species (**A**) and the corresponding color loci plotted in a hexagon color space against a standard green leaf (**B**). E_U_, E_B_, and E_G_ represent the excitation of the UV, blue, and green bee receptor, respectively. The pairwise distances between the color loci and between each color locus and the uncolored point (center of the hexagon) are given in the online Supplementary Material (Table S1).

With the exception of *C. thyrsoides*, which reflected light strongly from blue to red (450–700 nm), the flowers of all *Campanula* species had two peaks of reflectance, one in the violet-blue range (between 400–440 nm) and the other in the orange-red range (600–700 nm). Reflectance in the UV range (below 400nm) was stronger in some of the species (e.g., *C. latifolia*, *C. medium,* and *C. rapunculoides*) than in others (Figure 1A).

Color modelling revealed that the corolla color of most species appeared as either UV-blue (*C. glomerata*, *C. medium*, *C. latifolia*, *C. patula*, *C. rapunculoides*, *C. rapunculus*, and *C. scheuchzeri*) or blue (*C. moravica*, *C. persicifolia*, *C. punctata*, *C. rotundifolia*, and *C. trachelium*) to bees (Figure 1B). The only exceptions were *C. lactiflora*, whose flowers were at the intersection between the blue and the blue-green ranges, and *C. thyrsoides* with blue-green flowers. The distance of color loci between the species ranged from 0.03 (*C. medium* vs. *C. scheuchzeri*) to 0.13 (*C. medium* vs. *C. rapunculus*) hexagon units within the UV-blue category and from 0.02 (*C. persicifolia* vs. *C. rotundifolia*) to 0.09 (*C. punctata* vs. *C. trachelium*) within the blue category. The distance of color loci between the species of different bee color categories ranged from 0.07 (*C. moravica* vs. *C. patula*) to 0.52 (*C. glomerata* vs. *C. thyrsoides*). According to the model, the corolla color of all species was easily detectable by bees against a green standard background (i.e., Euclidean distances in the color hexagon were >0.1 units from the uncolored point; Table S1).

Multivariate analyses revealed significant differences in reflectance curves among species (ANOSIM: global R = 0.89, *p* < 0.001). Though most pairwise comparisons (75 of 91) yielded R-values higher than 0.75, pointing to a species-specific pattern of reflectance curves with only a very limited overlap among species, post-hoc analyses did not reveal significant differences between the reflectance curves of any pair of species, mainly because of low sample size, see [33] for a discussion of this topic. Among the other sixteen pairs of species, nine had R-values between 0.5 and 0.75, whereas seven had R-values below 0.5.

### 2.2. Comparisons of Floral Scent Bouquets among Campanula Species

In the scent bouquet of the various *Campanula* species, we detected 150 compounds, from which 102 compounds could be (tentatively) identified (Appendix A, Table A1; see Table S2 for a complete list of compounds). Representatives of seven compound classes were recorded in the samples: Monoterpenes (40 compounds), sesquiterpenes (30), aromatic compounds (12), aliphatic compounds (8), spiroacetals (6), N-compounds (4), and irregular terpenes (2). *Campanula glomerata* and *C. rotundifolia* had representatives from all compound classes, whereas *C. latifolia*, *C. persicifolia*, *C. rapunculoides*, *C. thyrsoides,* and *C. trachelium* from six classes, *C. rapunculus* from five classes, and *C. medium* and *C. punctata* from four classes. The number of floral volatile compounds produced by each species varied considerably, ranging from 10 in *C. punctata* to 55 in *C. trachelium*. None of the compounds were shared by all species. The compounds detected most frequently as floral scent constituents were 2-phenylethanol (10 species), (*E*)-conophthorin and 4-oxoisophorone (9 spp.), and (*E*)-β-caryophyllene (8 spp.). The scent bouquets of the species were generally dominated by a few compounds and the mean relative contribution to the total flower scent discharge of the five most abundant compounds of each species ranged from 43.1 ± 3.7% in *C. glomerata* to 93.3 ± 1.9% in *C. lactiflora*.

**Figure 2:**
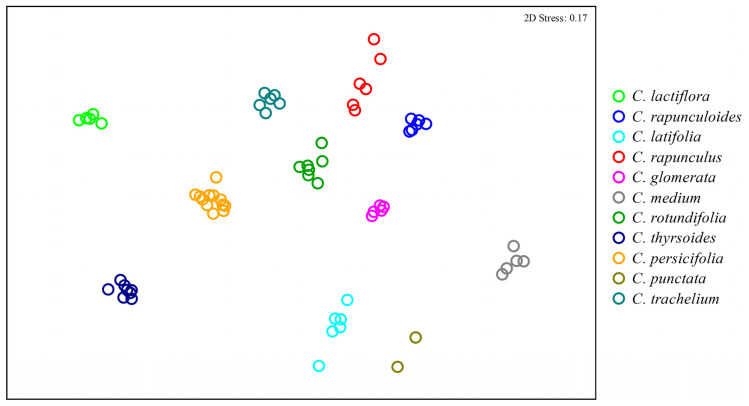
Comparison of floral scent bouquets among 11 *Campanula* species based on semi-quantitative Bray–Curtis similarities plotted with non-metric multidimensional scaling (NMDS). Samples grouped in a similar manner in the NMDS plot based on qualitative similarities (Figure S1, Supplementary Material).

Multivariate analyses revealed significant differences in both the qualitative (presence/absence of compounds; global R = 1, *p* < 0.001) and semi-quantitative (relative amount of compounds; global *R* = 1, *p* < 0.001) composition of floral scents among species. Post-hoc analyses revealed that composition differed significantly for all pairs of species (*R* = 1 for all pairs, *p* < 0.05 for both qualitative and semi-quantitative comparisons), showing that each species produced an exclusive scent profile (Figure 2) and that the sampling heterogeneity (i.e., number of samples, as well as individuals and flowers used for each species) has no effect on the analyses

### 2.3. Phylogenetic Signals in Floral Colour and Scent

**Figure 3:**
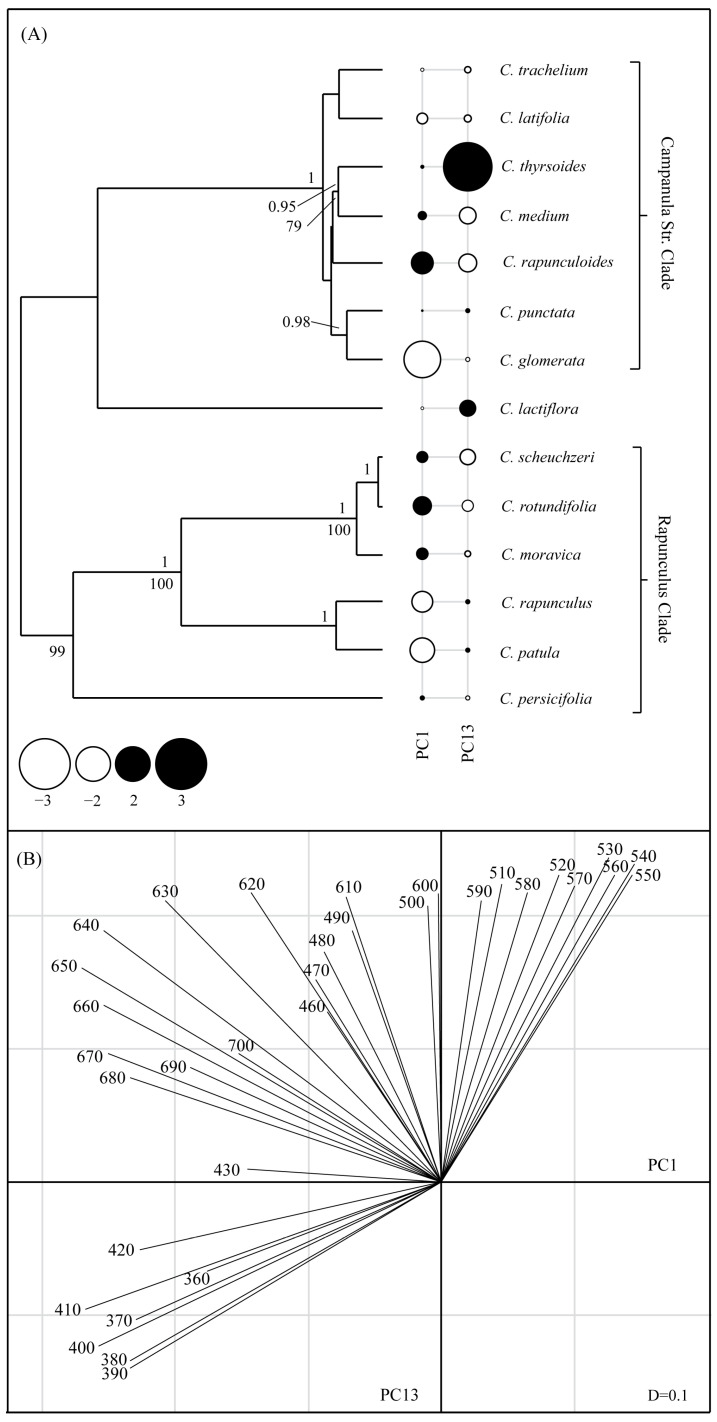
(**A**) Phylogeny of the species studied and results of the flower color pPCA. Positive and negative scores on PC1 (global) and PC13 (local) are indicated by black and white circles, respectively. Symbol sizes are proportional to absolute values. Bayesian posterior probabilities (BPP) ≥ 0.95 and ML bootstrap values ≥ 70% are indicated above and below branches, respectively. (**B**) Loading of the main traits for the global (horizontal axis) and local (vertical axis) principal components. Only the variables with highest loadings on the PCs are depicted. The loadings of all variables and scores of each species are given as Supplementary Material (Tables S3 and S4, respectively). D = 0.1 is the mesh of the grid.

**Figure 4:**
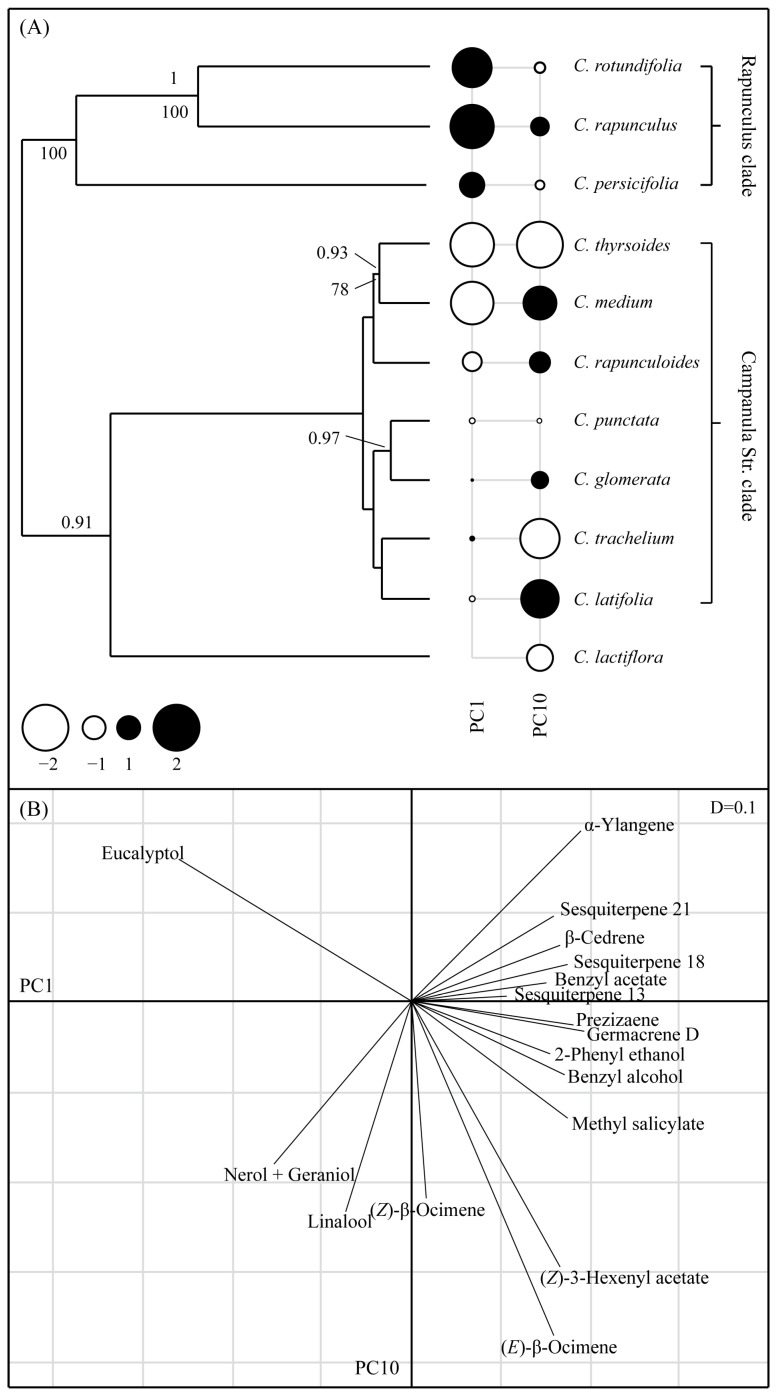
(**A**) Phylogeny of the species studied and results of the scent pPCA. Positive and negative scores on PC1 (global) and PC10 (local) are indicated by black and white circles, respectively. Symbol sizes are proportional to absolute values. Bayesian posterior probabilities (BPP) ≥ 0.95 and ML bootstrap values ≥ 70% are indicated above and below branches, respectively. (**B**) Loading of the main traits for the global (horizontal axis) and local (vertical axis) principal components. Only the compounds with the highest loadings on the PCs are depicted. The loadings of all variables and scores of each species are provided as Supplementary Material (Tables S5 and S6, respectively). D = 0.1 is the mesh of the grid.

Color and scent phylogenetic reconstructions by using ITS, petD, and trnL_F are depicted in Figure 3A and Figure 4A, respectively. Although the scent tree had three species fewer than the color tree, the structure of both trees was similar.

#### 2.3.1. Floral Color

We did not detect a phylogenetic signal in color variables by using Bloomberg’s K analysis (Kmult = 0.2, *p* = 0.26). Similarly, the global structures (PC1) of the pPCA did not show a phylogenetic signal, neither by the Abouheif test (C_mean_ = 0.16, *p* = 0.19) nor the Pagel’s λ test (λ = 0, *p* = 1). The lack of the signal in PC1 can be explained by the absence of a clear pattern in the loadings of the various wavelength reflectance values (Figure 3B). Both low (360–430 nm) and high (630–700 nm) wavelength reflectance had high negative loadings, whereas only wavelengths from 530-560 nm had medium values of positive loadings. As expected for local structures in a pPCA, no phylogenetic signal was apparent for PC13 (Abouheif test: C_mean_ = −0.22, *p* = 0.97; Pagel’s λ test: λ = 0, *p* = 1).

In the local structures (PC13), a clear pattern of the color variables was found: Wavelengths from 350 to 420 nm had negative loadings, whereas those from 450 to 640 nm had the highest positive loadings. The different reflectance properties in these ranges among some closely related species might be responsible for the absence of phylogenetic signals. For example, *C. medium* and *C. rapunculoides* had high reflectance between the 350 to 420 nm wavelengths, whereas closely related *C. thyrsoides* exhibited high reflectance between 450 and 640 nm. Another interesting aspect revealed by the pPCA analysis was the tendency of most pairs of closely related species to differ in their UV reflectance (mainly because of high or low reflectance between 350–400 nm). Among the five pairs of the most closely related species (*C. trachelium*/*C.latifolia*, *C. punctata*/*C. glomerata*, *C. scheuchzeri*/*C. rotundifolia*, *C. thyrsoides*/*C. medium,* and *C. rapunculus*/*C. patula*), only the latter did not diverge in color reflectance as seen through the bee eye. In this particular case, flowers of both species were UV-blue and the distance between color loci in the color hexagon was <0.1 (0.04). In all other pairs, flowers were either UV-blue or blue to bees and the distances between the color loci were always >0.1 (Table S1).

#### 2.3.2. Floral Scents

Using the multivariate Bloomberg’s K test, we detected no phylogenetic signal for the blend of all scent compounds (Kmult = 0.3, *p* = 0.73). However, the compounds that correlated most with the phylogeny (pPCA, Figure 4: Global structures; highest positive or negative loadings on PC1) showed a phylogenetic signal as assessed by both the Abouheif (C_mean_ = 0.71, *p* = 0.001) and Pagel’s λ (λ = 1, *p* = 0.03) tests. Four monoterpenes, i.e., eucalyptol, nerol, geraniol, and linalool had the highest negative loadings on PC1 (Figure 4B) and were characteristic for *C. thyrsoides, C. medium* and *C. rapunculoides*, which clustered in a clade and had the highest negative scores (PC1, Figure 4). Sesquiterpenes, such as α-ylangene, prezizaene, β-cedrene, and germacrene D, and aromatics, such as 2-phenyl ethanol, benzyl acetate, benzyl alcohol, and methyl salicylate, had positive loadings on PC1. The three species included in the *Rapunculus* clade (Figure 4) had the highest positive scores, which were generated by the presence of 2-phenyl ethanol, α-ylangene, prezizaene, and germacrene D. The two species with the highest positive scores (*C. rodundifolia* and *C. rapunculus*) also shared benzyl acetate, benzyl alcohol, methyl salicylate, and β-cedrene. The two species with the highest negatives scores (*C. thyrsoides* and *C. medium*) were the only species that produced eucalyptol. Nerol and geraniol were only produced by *C. thyrsoides*. Sesquiterpenes with high positive loadings (prezizaene, β-cedrene, and germacrene D) were absent from the scent bouquet of *C. rapunculoides*, *C. thyrsoides,* and *C. medium*.

Similar to the results of the pPCA concerning corolla reflectance, neither the Abouheif (C_mean_ = −0.38, *p* = 0.997) nor the Pagel’s λ (λ = 0, *p* = 1) test detected a phylogenetic signal in the local structures (PC10). In the latter structures, eucalyptol and α-ylangene had the highest positive loadings, with (*Z*)-3-hexenyl acetate and (*E*)-β-ocimene having high negative loadings (PC10, Figure 4B). The compounds with high (positive or negative) loading values in the PC10 accounted for differences between closely related species. For example, (*E*)- and (*Z*)-β-ocimene, linalool, nerol, and geraniol were found in high relative amounts in the flowers of *C. thyrsoides* but not of *C. medium*, whereas eucalyptol was produced in high relative amounts in flowers of *C. medium*, but not in flowers of *C. thyrsoides.*

## 3. Discussion

In the present study, we compared floral color and scent of 14 and 11 *Campanula* species, respectively, and gained insights into the different evolutionary paths of these floral cues within this genus. We found evidence of a phylogenetic signal for some floral scent constituents (Abouheif’s C mean and Pagel’s λ indices showed signal in pPCA global structures), but not for others and for the overall scent bouquet. In contrast, for floral reflectance properties, we found no evidence of phylogenetic signal at all. While the detection of phylogenetic signals in some constituents of the scent bouquet of *Campanula* species suggests that the evolution of scents in this genus is constrained in part by phylogenetic relatedness, the lack of phylogenetic signals in other scent constituents and in the whole scent bouquet, as well as in color reflectance, all point to external agents (e.g., pollinators) shaping the evolution of those traits. The occurrence of both bee UV-blue and bee blue colors within the different clades suggests that selection has favored these two colors in the genus *Campanula*. Indeed, the innate preference that many bee species display to each of these colors (see discussion below) indicates that the evolution of color in *Campanula* has been mainly shaped by the visual preferences of the pollinating bees. Taken together, our results suggest that the visual and olfactory biases of pollinating bees exert unequal selective pressures on floral color and scent of a bee-pollinated genus, as supported by the different extent in which these cues are influenced by phylogenetic relatedness.

### 3.1. Floral Colour and Its Evolution in Campanula

The color analyses performed in this study revealed that the corolla of most *Campanula* species is either UV-blue or blue to bees. Our results are congruent with those from 14 further *Campanula* species available in the floral reflectance database FReD [34]; indicating that UV-blue and blue are well-established bee colors in this genus. The lack of phylogenetic signal in the corolla color reflectance of the *Campanula* species investigated here, together with the widespread occurrence of both UV-blue and blue bee colors across the phylogeny, suggest that these colors either have been repeatedly selected (possibly as a response to the pre-existing visual biases of pollinating bees, as speculated previously for other bee-pollinated plants [35,36]) or represent, in this genus, a plesiomorphic trait that has been retained through stabilizing selection.

Color is crucial in the flower location of bees in general [11] and phenotypic variations in this floral trait are believed to be driven by the innate visual biases of pollinators [17]. *Campanula* species are pollinated by a vast array of bees [22,23], meaning that the differential visual biases of the bee species might shape the evolution of color in this genus. Innate color preference in bees has been investigated for only a few species, including some polylectic species of the genus *Bombus* [37,38] and *Apis mellifera* [39] and the oligolectic *Chelostoma rapunculi* [40], *Ch. campanularum,* and *Hoplitis mitis* (Milet-Pinheiro et al. unpublished data). These species, all frequent visitors/pollinators of *Campanula*, present an innate preference either for blue or UV-blue colors. The UV-blue and blue bee color categories might thus be an adaptive floral trait in the genus *Campanula*. Further experimental studies of the innate color preference of other frequent pollinators of *Campanula* will help testing this assumption.

The most divergent species in terms of color was *C. thyrsoides*. This species clustered in a well-supported clade with *C. medium* and *C. rapunculoides.* Its color differed considerably from the colors of these closely related species, mainly because of high corolla reflectance at between 450 and 650nm. This result is surprising, since *Campanula thyrsoides* is pollinated mainly by bumblebees [41]. As mentioned above, bumblebees often display an innate preference for violet-blue colors. However, several recent studies have found that innate color preference diverges between different bumblebee populations [37,38,42,43,44], suggesting that local variation in floral traits drives selection of innate color preferences. *Campanula thyrsoides* occurs in sub-alpine and alpine grasslands, typically between 1600m to 2200m a.s.l. [41], where bee blue-green and green colors are more representative in plant communities [45], but see [46,47]. In such a floral market, bees with a stronger visual bias for bee blue-green and green colors (over UV-blue or blue) should forage more efficiently, whereas an innate preference for UV-blue/blue could be maladaptive. Unfortunately, to the best of our knowledge, the innate color preference of alpine bumblebee populations has not previously been investigated. Nevertheless, the innate preference for yellow over blue (and vice-versa) in some colonies of the North American bumble bee *Bombus impatiens* [42] suggests that the evolution of an innate preference for yellow in alpine bumblebee populations is possible.

### 3.2. Floral Scent Composition and Its Evolution in Campanula

The absence of phylogenetic signals on multivariate scent composition suggests that the evolution of floral scents in *Campanula* is shaped by external selective agents (e.g., pollinators), whereas the presence of a phylogenetic signal in some specific compounds suggests that scent bouquet is partly constrained by phylogenetic relatedness. These apparently contrasting results indicate that different compounds within the flower scent blend have different evolutionary histories that result in some compounds mirroring phylogenetic relatedness and others not [48]. Similar findings have also been reported for the bee-pollinated genus *Lysimachia* (Primulaceae). In this study, Schäffler and colleagues [20] investigated oil-secreting and non-oil-secreting species, pollinated by specialist oil-collecting bees and generalist pollen/nectar-seeking bees, respectively. They found evidence that some compounds were correlated with phylogeny, whereas others had evolved in a convergent manner, presumably as a result of pollinator-mediated selection. The compounds that were assumed to be under pollinator-mediated selection were later shown to be involved in the recognition of the oil-secreting flowers of *Lysimachia* by oil-collecting *Macropis* bees [49]. Further evidence of pollinator-mediated selection comes mainly from other specialized pollination systems in which unrelated plants depend on a single or a few related pollinator species (as is the case for oil-secreting plants). In these systems, floral scent composition usually converges as a response to the very specific innate olfactory preference of a subset of related pollinators [6,50,51,52,53]. In the case of *Campanula* species, the possible conflicting scent preferences of the different unrelated pollinating bee species [32] might have loosened any specific and directional selective pressures on floral scents, as supported by our findings of strong phylogenetic signals in compounds that correlated most with the phylogeny. However, in cases in which the distinct scent preferences of various bee species exert additive selective pressures [27], scent might be more shaped by the overall preferences of the bees.

The apparent influence of phylogeny on some floral scent components in *Campanula*, however, should not be interpreted as evidence that external agents, such as pollinators (and possibly herbivores, herbivores’ natural enemies, and pathogenic microorganisms), play an irrelevant role in shaping the evolution of these phylogenetic constrained compounds. As revealed by the pPCA analysis, several of the volatiles most responsible for the global (e.g., benzaldehyde, eucalyptol 2-phenyl ethanol, nerol, and geraniol) and local structures (e.g., eucalyptol, (*Z*)-3-hexenyl acetate, (*Z*)- and (*E*)-β-ocimene, linalool, nerol, and geraniol) are not only known as potent attractants of several bee species [14,54,55,56,57,58], but also as a repellent/deterrent of herbivores [59,60,61,62,63], as an attractant of herbivores natural enemies [64,65], and as a growth inhibitor of pathogenic microorganisms [66,67,68]. This provides a strong indication for natural selection on floral scents of *Campanula* having fostered compounds that are attractive to mutualists and/or repellent/detrimental to antagonists.

As expected, we did not detect phylogenetic signals in local structures. This reflects the highly discrepant floral scent composition reported for closely related species, as in the case of *C. latifolia* and *C. trachelium*. The discrepancy between the floral scents of these two species is generated mainly by their major compounds. α-Ylangene is the main compound of *C. latifolia* (>60% of the total relative scent bouquet) but is absent in *C. trachelium*. In contrast, (*Z*)- and (*E*)-β-ocimene are the major compounds of *C. trachelium* (>40%) but are absent in *C. latifolia*. The same rationale, involving different compounds, is true for other pairs of closely related species investigated here, suggesting that scents are evolutionary flexible in the genus *Campanula*. Floral scents are known to act as a primary mechanism of floral isolation in many plant–pollinator interactions, by selectively attracting a specific subset of flower-visiting insects in a given community or simply by changing the relative frequency of some pollinator species in flower visitor assemblages [53,69,70,71]. Some of the closely related species investigated here, such as *C. trachelium* and *C. latifolia,* and *C. rotundifolia* and *C. rapunculus*, occur sympatrically, have similar flowering periods, and share several bee species as pollinator. Therefore, the striking divergence in floral scents of closely related *Campanula* species might assure a differential attraction of some bee species of the pollinator assemblages, through species-specific olfactory preferences, for example [72], thereby acting as a pre-mating reproductive barrier. The forces selecting such discrepant floral scents between closely related species seem to be particularly strong, because other floral filters, which are known to promote pollinator partitioning, are absent. For example, the flower morphology of the closely related *Campanula* species is rather similar [24] and might not filter for the different subsets of potential pollinating bees, see, for example, [73].

The multivariate statistical comparisons revealed that each *Campanula* species has a unique scent profile. Among angiosperms, specificity in floral scents seems to be the general rule e.g., [20,74,75,76,77], suggesting that stabilizing selection operates on floral scents within species for some exceptions, see [78,79]. This specificity is normally assumed to be adaptive because strong differences in floral scents of conspecific individuals can result in the attraction of distinct pollinator species, thereby leading to reproductive failure [79]. Furthermore, many pollinators (including bees) can learn to discriminate subtle differences in scent bouquets by associative learning with the availability of a reward, such as pollen and nectar [57,80,81]. Thus, even minor differences among floral scent bouquets can promote floral constancy by pollinators, with the benefit of reducing both pollen loss and inter-specific pollen flow when bees visit flowers of other species [82,83]. Specificity in floral scents might therefore play a crucial role as an isolating mechanism in the genus *Campanula*, mainly because other common isolating mechanisms, such as flowering phenology and floral morphology, are less relevant.

## 4. Materials and Methods

### 4.1. Study Species

**Figure 5:**
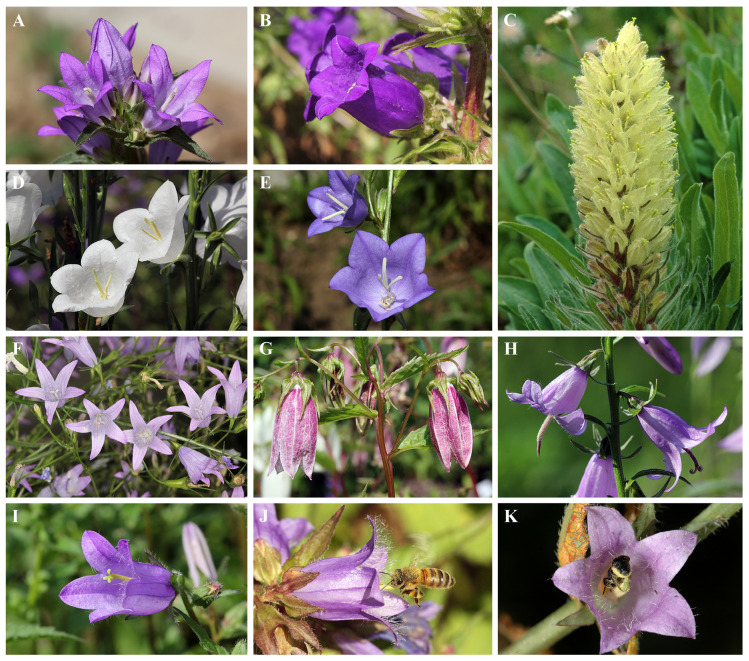
Flowers and flower visitors of some of the *Campanula* species investigated in this study. (**A**) *C. glomerata*, (**B**) *C. medium*, (**C**) *C. thyrsoides*, (**D**) *C. persicifolia* subsp. *alba*, (**E**) *C. persicifolia*, (**F**) *C. rapunculus*, (**G**) *C. punctata*, (**H**) *C. rapunculoides,* and (**I**) *C. trachelium*, (**J**) a honey bee worker inspecting a flower of *C. trachelium* and (**K**) pollen-specialist *Chelostoma rapunculi* female gathering pollen on *C. trachelium*. All photographs by Paulo Milet-Pinheiro, except for *C. thrysoides* (**C**), by Heiko Bellmann.

We assessed the evolution of floral color and scent in 14 and 11 *Campanula* species, respectively (Table 1, Figure 5). We integrated the information of our previous studies of the color and scent of six *Campanula* species [40,84] with those collected in the present study (Table 1).

Plant individuals of the five species used for scent sampling in the present study (Table 1) were cultivated to the seedling stage in the greenhouses of the Department of Plant Systematics, University of Bayreuth, and of the Botanical Garden of Ulm, University of Ulm. Subsequently, plants were cultivated outdoors in flowerbeds, where they grew to maturity. The color reflectance properties of *C. moravica*, *C. patula,* and *C. scheuchzeri* were measured in a few individuals cultivated in fields at the Ecological-Botanical Garden of the University of Bayreuth. Unfortunately, we were not able to additionally collect scent samples from these three species because only few individuals were available.

### 4.2. Colour Measurements of Campanula Flowers and Bee Colour Hexagon

The corolla reflectance properties of *Campanula* taxa were recorded from 300 nm to 700 nm the wavelength perceived by bees [15]; by using a Varian Cary 5 spectrophotometer equipped with a Praying Mantis accessory (Varian, Inc., Palo Alto, CA, USA), as used in our previous investigations into *Campanula* color [40]. For each species, measurements were taken from the inner surface of the corolla of three plant individuals (one flower per individual). For each flower, two measurements were taken, one from the base and the other from the tip of the corolla. We used only recently opened flowers to avoid possible influence of flower ageing/pollination in reflectance properties. Barium sulphate was used as a white standard and the disconnected beam as the black reference.

The mean reflectance of the petals (composed first from the measurements of the tip and base of each flower, and then from the three replicates per species) were used for the phylogenetic PCA analysis (see below) and to determine the loci of corolla colors in the hexagon color space [87]. We applied the daylight irradiance spectrum D65 as a standard and used the spectral sensitivity of the photoreceptors of the honeybee as a representative approximation for *Campanula*-visiting bees [88] given that bees normally do not differ substantially in their visual sensory systems [15]. The reflectance function of a typical green leaf was used as the background color [89]. The position of the color loci indicate the way that bees perceive the corollas through their UV, blue, and green photoreceptors and via further processing of receptor signals in the central nervous system [43]. The bee hexagon is divided into six segments, each one corresponding to different bee-color categories, i.e., UV, UV-blue, blue, blue-green, green, UV-green [89].

For a comparison of bee colors among the various *Campanula* species, we calculated the pairwise hexagon distances of color loci among species and the distance of each color locus to its background (green leaves) [88]. Behavioral experiments with bumblebees trained to visit artificial flowers have demonstrated that color distances <0.05 hexagon units and >0.10 hexagon units are poorly and easily discriminated, respectively [90].

### 4.3. Sampling of Floral Scents

In order to obtain scent samples for the chemical analyses, volatiles were collected from inflorescences by using standard dynamic headspace methods, following our previously described protocol [40]. The number of samples and flowers used varied considerably among species (Table 1), according to the availability of individuals, as well as the different flowering behavior (i.e., different number of flowers per inflorescences) of each species. For every sample, the inflorescences in full bloom from two to three different plants were enclosed together in a polyester oven bag (20 × 30 cm; Toppits^®^). The volatiles were then trapped for 4 h in an adsorbent tube through which air was drawn at a rate of 200 mL min^−1^ by using a membrane pump (G12/01 EB, Rietschle Thomas, Puchheim, Germany). The adsorbent tubes consisted of ChromatoProbe quartz microvials (GC/MS: length: 15 mm; inner diameter: 2 mm; Varian Inc., Palo Alto, CA, USA), cut at the closed end and filled with a mixture of 1.5 mg Tenax-TA (mesh 60–80; Supelco, Bellefonte, PA, USA) and 1.5 mg Carbotrap B (mesh 20–40, Supelco, Bellefonte, PA, USA), which was held in the tubes by using glass wool [91]. Headspace samples of non-flowering plants (*n* = 3 per species) were also collected following the aforementioned methods in order to control for non-floral (vegetative) volatiles and ambient contaminants in the floral scent samples. All headspace samples were stored in screw cap vials at −20 °C until chemical analysis.

### 4.4. Chemical Characterization of Floral Scents

In order to characterize the flower scent chemistry of *Campanula* taxa, the headspace samples were analyzed on a Varian Saturn 2000 mass spectrometer coupled to a Varian 3800 gas chromatograph (GC/MS) equipped with a 1079 injector (Varian Inc., Palo Alto, CA, USA), which had been fitted with the ChromatoProbe kit [see 91] and a fused silica column ZB-5 (5% phenyl polysiloxane; 60 m long, inner diameter 0.25 mm, film thickness 0.25 µm, Phenomenex). Each quartz microvial was loaded into the probe, which was then inserted into the modified GC injector. Equipment configurations were identical to those published elsewhere [40].

Identification of compounds was carried out by using the NIST 08, Wiley 7 or Adams [92] mass spectral databases or the database provided in MassFinder 3 and were confirmed by a comparison of retention times with published data [92]. The structure assignments of individual components were confirmed by a comparison of mass spectra and GC retention times with those of available standards.

The composition of volatiles collected from flowering plants was compared with that from non-flowering plants (vegetative parts). Floral scent volatiles were those detected either at higher amounts (i.e., peak areas in chromatrogram of floral scent samples at least 10-fold larger than in leave samples) or exclusively in floral scent samples.

### 4.5. Statistical Analyses of Floral Colour and Scent

Differences in the reflectance curves of the investigated *Campanula* species were tested by using the multivariate analysis of similarity (ANOSIM). For this, we first standardized curves in relation to the maximum reflectance of each species, i.e., the highest reflectance was set at 100%, whereas the reflectance at the other wavelengths was adjusted accordingly. We then created a resemblance matrix based on Euclidean distances to assess pairwise distances among individual samples. We used the obtained similarity matrix to run an ANOSIM analysis (10,000 permutations) and to graphically display each sample in a plot of non-metric multidimensional scaling (NMDS). ANOSIM operates directly on a (dis)similarity/distance matrix. It yields a test statistic R that is a relative measure of separation among a priori defined groups and is based on differences of mean ranks among and within groups. An R value of zero indicates completely random grouping, whereas a value of one indicates that samples within groups are more similar to each other than to any sample from a different group [33].

Scent data of the different species were compared by using qualitative (presence/absence of compounds) and semi-quantitative (relative amount of each compound with respect to total peak area transformed to their fourth root) approaches. For the analyses of qualitative and semi-quantitative differences in floral scent bouquet among species, we calculated Sørensen and Bray-Curtis similarity indices, respectively, to assess pairwise similarities among the individual samples. As for color, we then used the similarity matrices to run ANOSIM and NMDS analyses. The software Primer 6.1.6 was used to calculate the similarity indices and to perform the ANOSIM and NMDS analyses [33].

### 4.6. Phylogenetic Signals in Colour and Scent

#### 4.6.1. Phylogenetic Reconstructions

In order to detect possible phylogenetic constraints in color reflectance and scent composition, we investigated the phylogenetic signal of both floral traits. First, we generated two phylogenetic trees, namely “color” (species with available color data) and “scent” (species with available scent data). The “color” and “scent” trees included 14 and 11 terminal taxa, respectively (Table 1). Although the scent tree had three species fewer than the color tree, the structure of both trees was similar (see Results). In order to generate the phylogenetic trees, we employed the chloroplastic petD protein (petD) gene, the trnL-trnF intergenic spacer (trnL-F), and the internal transcribed spacer complete repeat (ITS) of the ribosomal DNA as genetic markers, all available in GenBank (Table 1). Sequences were aligned by using the multiple sequence alignment tool MAFFT v7 [93]. Major insertions and ambiguous regions in the alignments were identified and eliminated with Gblocks version 0.91b [94] by means of the relaxed parameter values suggested by Talavera and Castresana [95]. We used maximum likelihood (ML) and Bayesian inference (BI) for the phylogenetic analyses of the combined dataset of petD, trnL-F, and ITS. We performed ML analyses in RAxMLGUI 1.3, which is a graphical front-end for RAxML Randomized Accelerated Maximum Likelihood for High Performance Computing [96]. We then partitioned the dataset by locus and performed 10 runs, assessing node support via 10,000 bootstrap replicates. Bayesian analyses [97] were accomplished with the software MrBayes 3.2.1 [98]. We selected the model of nucleotide substitution that fitted best for every particular partition, according to the Akaike Information Criterion (AIC) in jModeltest [99]. Selection of priors and convergence assessment was achieved following Millanes et al. [100]. The ultrametric trees used in the pPCA analyses (see below) were generated under a Bayesian approach by using BEAST version 1.7.5 [101].

#### 4.6.2. Phylogenetic Signal and Phylogenetic Principal Component Analyses (pPCA)

The phylogenetic signal is the tendency of related species to resemble each other in any parameter [102]. Phylogenetic principal component analysis, hereafter pPCA [103], uncovers the phylogenetic signal in multivariate sets of traits and also the variables that create similarities/dissimilarities among closely related species. To test for phylogenetic signals of floral color and scent, we performed two independent pPCA analyses. For this purpose, we constructed two matrices, one based on the color reflectance data and the other on the semi-quantitative scent composition. In the color matrix, the corolla reflectance value every 10 nm (between 300 and 700 nm) was considered as one variable. In the scent matrix, each compound was considered as one variable. In the pPCA analyses, the phylogenetic resemblance in a complex set of continuous variables is graphically summarized into two principal components (PCs) [103]. The first PC denotes the global structure and reveals the variables that are more similar in related than in distant species (highest phylogenetic signal). The local structure is depicted in the second PC, which reveals the variables that create dissimilarities among closely related species (lowest phylogenetic signal). Phylogenetic principal component analysis was implemented with the adephylo package [104] in the R statistical environment [105]. In the pPCA, we used the measure of phylogenetic proximity underlying the test of Abouheif [106] because of its ability to detect phylogenetic signals [107]. In each PC, score values (positive or negative) are assigned to each species in the tree. Positive or negative scores denote larger values in the variables with more importance in the positive or negative PC sides, respectively. As pPCA does not explicitly test for the presence of a phylogenetic signal, we tested the signal in the scores of the PCs. We used Abouheif’s C mean (999 permutations) and Pagel’s λ because they performed better than other indices under a Brownian motion model [108]. As the variables that correlate the most and least with the phylogeny are in the global and local structures, respectively, we expected significant phylogenetic signal in the global but not in the local structures. Finally, to test for the significance of the phylogenetic signal in multivariate color reflectance and scent data traits, we used the multivariate Bloomberg’s K Kmult; [109]. Kmult ≈ 1 indicates evolution of traits by Brownian motion. We estimated Abouheif’s C mean with the function abouheif.moran implemented in adephylo [104], Pagel’s λ by using the function fitContinous of the package geieger [110], and Kmult with the R script available in [109].

## Figures and Tables

**Table 1:** List of *Campanula* species investigated for corolla color reflectance and floral scents, with their respective native occurrence. Number of scent samples and GenBank accession numbers of sequences used for testing phylogenetic patterns are given. For color analyses, N = 3 for all species. References: (A) Rosenbauer [85]; (B) Kovacic [21]; (C) Inoue and Amano [86]; and (D) Kuss et al. [41].

*Campanula* Species	Native Occurrence	Color Analyses	Scent Analyses (No. of Samples; No of Individuals; Total No. of Flowers)	GenBank Accession Number (petD/trnL–F/ITS)
*C. glomerata* L.	Eurasia (A)	Yes *	Yes * (5; 15; 235)	JX914747/HQ704671/HQ704552
*C. lactiflora* M. Bieb	Turkey and Caucasus (B)	Yes *	Yes * (5; 10; 300)	JX915131/FJ589212/-
*C. latifolia* L.	Eurasia (A)	Yes	Yes (6; 12; 139)	JX914791/EF088732/-
*C. medium* L.	South Europe (B)	Yes	Yes (5; 10; 63)	FN397024/EF088738/HQ823432
*C. moravica* (Spitzn.) Kovanda	Europe	Yes	No	-/EF088740/-
*C. patula* L.	Europe (A)	Yes	No	JX914974/EF213148/FM212739
*C. persicifolia* L.	Europe (A)	Yes *	Yes * (12; 12; 669)	JX915226/EF088743/DQ304590
*C. punctata* Lam.	Asia (C)	Yes	Yes (2; 6; 24)	JX915031/EF088753/HQ704550
*C. rapunculoides* L.	Eurasia (A)	Yes *	Yes * (6; 18; 451)	JX915191/EF088757/HQ823434
*C. rapunculus* L.	Europe/North Africa (A)	Yes	Yes (6; 18; 1049)	JX914708/EF088758/FM212738
*C. rotundifolia* L.	Circumpolar (A)	Yes *	Yes * (7; 18; 418)	JX915164/EF088759/DQ304615
*C. scheuchzeri* Vill.	Europe (A)	Yes	No	JX915162/EF088762/DQ304614
*C. thyrsoides* L.	Europe (D)	Yes	Yes (8; 16; 154)	FN397046/KJ512699/DQ304575
*C. trachelium* L.	Eurasia (A)	Yes *	Yes ** (6; 18; 379)	JX914678/EF088774/DQ304572

* Data concerning flower reflectance properties and scent from Milet-Pinheiro et al. [40]. ** Data concerning flower scent from Milet-Pinheiro et al. [84].

## Data Availability

The data presented in this study are available as Supplementary Material.

## Supplementary Materials

The following are available online at https://www.mdpi.com/article/10.3390/plants10071356/s1, Figure S1: Comparison of floral scent bouquets among 11 *Campanula* species based on qualitative Sørensen similarities plotted with non-metric multidimensional scaling (NMDS), Table S1: Euclidean distances (in hexagon units) among flower color loci of *Campanula* species, Table S2: Number and mean percentage amount of volatile compounds in scent samples collected from *Campanula* inflorescences of 11 species, Table S3: Loading of compounds in the PCs of the color phylogenetic PCA (pPCA), Table S4: Scores of *Campanula* species in the PCs of the color Phylogenetic PCA (pPCA), Table S5: Loading of wavelengths (300–700 nm) in the PCs of the scent phylogenetic PCA (pPCA), Table S6: Scores of each *Campanula* species in the PCs of the scent phylogenetic PCA (pPCA).

## Appendix A

**Table A1:** Number and mean percentage amount of volatile compounds in scent samples collected from *Campanula* inflorescences of 11 species: *Campanula glomerata* (GLO), *C. lactiflora* (LAC), *C. latifolia* (LAT), *C. medium* (MED), *C. persicifolia* (PER), *C. punctata* (PUN), *C. rapunculoides* (RPC), *C. rapunculus* (RAP), *C. rotundifolia* (ROT), *C. thyrsoides* (THY), and *C. trachelium* (TRA). Volatiles are listed according to chemical class and Kovats Retention Indices (KI). The number of compounds not identified and the percentage of amount of scent not identified are also given.

Compound Name	KI	GLO	LAC	LAT	MED	PER	PUN	RPC	RAP	ROT	THY	TRA
No. of compounds		43	32	30	15	28	10	30	33	41	46	55
*Aliphatics*												
(*E*)-2-Hexenal *	855	-	-	-	-		-	-	-	-	0.88	-
(*Z*)-3-Hexenol *	857	-	-	-	-		-	-	-	-	4.14	-
(*E*)-2-Hexenol	867	-	-	-	-		-	-	-	-	1.55	-
Hexanol *	868	-	-	-	-		-	-	-	-	1.21	-
(*Z*)-3-Hexenyl acetate *	1006	12.99	12.24	-	-		48.60	-	-	55.54	-	16.70
Hexyl acetate *	1013	-	0.13	-	-		28.93	-	-	2.64	-	-
2-Nonanone *	1094	-	-	-	-		-	-	-	-	-	1.26
Tridecane *	1300	-	-	-	-		-	-	-	-	-	4.61
*Aromatics*												
*p*-Methylanisole *	1025	0.70	-	2.92	-		-	-	-	-	-	-
Benzyl alcohol *	1039	-	-	-	-		8.03	-	6.71	2.30	0.33	-
Phenylacetaldehyde *	1050	-	-	-	-	2.42	-	61.71	41.13	-	-	3.40
1-Phenylethanol	1066	-	-	-	-		-	-	-	0.46	-	-
*o*-Guaiacol *	1095	0.33	-	0.23	-		-	-	-	-	-	0.06
2-Phenylethanol *	1121	4.29	0.11	0.75	-	5.58	6.76	9.87	9.85	2.68	0.20	4.60
Benzyl acetate *	1169	-	-	-	-		-	-	1.30	0.25	-	-
Methyl salicylate *	1209	5.39	-	-	-		-	-	8.62	8.75	1.82	0.75
*o*-Anisaldehyde	1253	0.68	-	-	-		-	-	-	-	-	-
2-Phenylethyl acetate *	1263	-	-	-	-		-	0.31	0.80	0.25	-	0.10
*p*-Anisaldehyde *	1266	0.97	-	-	-		-	0.61	-	-	-	-
4-Ethylguaiacol	1287	-	-	-	-		-	-	-	0.10	-	-
*Irregular terpenes*												
4-Oxoisophorone *	1151	1.37	0.03	0.31	1.90	0.16	-	0.47	0.59	0.06	0.12	-
Geranyl acetone *	1460	-	-	-	4.05		-	-	-	-	-	0.24
*Monoterpenes*												
Artemisiatriene	928	-	-	-	-		-	-	-	-	0.03	-
α-Pinene *	942	-	-	-	-	2.24	-	-	-	-	-	-
Sabinene *	982	5.09	-	0.51	-	1.10	-	-	-	-	0.47	-
β-Pinene *	985	-	-	-	-	0.57	-	-	-	-	-	-
β-Myrcene *	995	-	0.51	2.14	-	8.52	-	-	-	0.45	7.01	-
α-Phellandrene *	1010	-	-	-	-		-	-	-	-	0.38	-
δ-3-Carene *	1018	1.06	-	1.24	-		-	-	-	-	-	0.27
α-Terpinene *	1027	-	-	-	2.81		-	-	-	-	0.35	-
Limonene *	1036	-	-	-	-		-	-	-	-	3.77	-
β-Phellandrene *	1038	-	-	-	-		-	-	-	-	0.48	-
(*Z*)-β-Ocimene *	1040	-	20.26	-	-	8.18	-	-	-	-	1.60	4.16
Eucalyptol *	1041	-	-	-	69.29		-	-	-	-	0.24	-
(*E*)-β-Ocimene *	1052	-	59.69	-	-	24.60	-	-	-	7.33	4.40	38.57
γ-Terpinene *	1065	-	-	-	-		-	-	-	-	0.23	-
(*Z*)-Linalool oxide (furanoid) *	1079	-	-	-	-		-	-	-	-	0.09	-
(*E*)-Linalol oxide (furanoid) *	1094	-	-	-	-	1.31	-	9.67	-	-	1.03	0.05
Terpinolene *	1096	0.46	-	-	-	0.32	-	-	-	-	0.93	0.07
Linalool *	1103	-	-	-	-	1.57	-	-	-	-	10.30	1.95
Allo-ocimene *	1132	-	0.64	-	-	3.60	-	-	-	-	0.55	-
p-Cymene *	1133	-	-	-	-		-	-	-	-	-	0.43
(*E*)-Epoxyocimene *	1144	-	0.08	-	-	0.33	-	-	-	-	-	0.28
Neo-allo-ocimene *	1145	-	0.02	-	-	0.27	-	-	-	-	0.04	-
Citronellal *	1156	-	-	-	-		-	-	-	-	0.10	-
Nerol oxide	1159	-	-	-	-		-	-	-	-	0.11	-
Dill ether	1162	-	0.15	-	-		-	-	-	-	0.24	-
(Z)-Isocitral	1167	-	-	-	-		-	-	-	-	0.09	-
Lavandulol	1172	-	-	2.19	-		-	-	-	-	1.19	-
p-Mentha-1,5-dien-8-ol	1176	-	0.09	-	-		-	-	-	-	-	-
(*E*)-Linalool oxide (pyranoid)	1181	-	-	-	-		-	4.21	-	-	0.04	-
(*E*)-Isocitral	1185	-	-	-	-		-	-	-	-	0.18	-
α-Terpineol *	1203	0.63	-	-	-	1.78	-	-	0.38	0.20	1.53	0.03
Verbenone *	1227	-	-	-	-		-	-	-	-	-	0.05
β-Citronellol *	1230	-	-	-	-		-	-	-	-	0.77	-
Nerol *	1234	-	-	-	-		-	-	-	-	22.86	-
(*Z*)-Ocimenone	1237	-	0.06	-	-		-	-	-	-	-	-
Neral *	1247	-	-	-	-		-	-	-	-	2.22	-
Geraniol *	1258	-	-	-	-		-	-	-	-	22.21	-
Geranial *	1276	-	-	-	-		-	-	-	-	2.69	-
Lavandulyl acetate *	1289	0.17	-	0.87	0.54		-	-	0.03	0.41	0.65	-
Methyl geranate *	1326	-	-	-	-		-	-	-	-	0.05	-
*N-compounds*												
Benzeneacetonitrile	1147	1.69	-	3.80	-	0.59	3.08	0.72	-	-	-	-
Indole *	1304	-	-	-	-		-	0.67	0.04	0.11	-	-
1-Nitro-2-phenyl ethane	1309	0.71	-	0.70	-		0.39	0.18	-	-	-	-
o-Aminoacetophenone	1313	-	0.37	-	-		-	0.23	0.12	-	-	-
*Sesquiterpenes*												
α-Cubebene	1365	1.59	-	-	0.86		-	0.40	0.09	0.07	-	0.23
α-Longipinene	1379	-	-	-	-	0.48	-	-	-	-	-	0.12
α-Ylangene	1389	2.04	-	68.8	-	10.78	-	2.00	0.61	1.43	-	0.68
α-Copaene *	1395	6.47	-	0.63	2.73		-	1.17	0.16	0.69	-	0.34
Longifolene *	1402	-	-	-	-		-	-	-	-	-	0.08
β-Bourbonene	1407	-	-	-	2.13	0.67	-	1.67	3.05	0.94	-	-
β-Elemene	1407	-	-	-	-		-	-	-	-	-	1.99
Ylanga-2,4(15)-diene	1422	-	-	0.21	-		-	-	-	-	-	-
β-Cedrene	1441	0.52	-	1.33	-		-	-	7.57	0.22	-	0.59
(*E*)-β-Caryophyllene *	1445	5.16	-	0.56	2.30	11.72	-	-	2.77	7.08	0.85	0.64
(*E*)-α-Bergamotene	1454	-	-	-	-		-	-	-	-	-	2.08
β-Gurjunene	1460	-	-	-	-		-	-	0.20	-	-	-
(*E*)-β-Farnesene *	1460	2.05	0.30	-	-		-	0.50	0.31	0.08	-	1.79
α-Caryophyllene *	1480	0.45	0.18	0.11	-		-	-	-	1.51	-	-
Prezizaene	1484	-	-	-	-	0.62	-	-	3.60	0.03	-	1.58
Amorpha-4,11-diene	1485	-	-	-	-		-	-	-	-	-	0.80
Allo-aromadendrene	1488	0.64	-	-	1.15		-	0.43	0.05	0.09	-	-
β-Cubenene	1493	0.12	-	-	-		-	0.13	-	-	-	-
Ar-Curcumene	1494	-	-	-	-		-	-	0.11	Tr	-	0.95
γ-Muurolene	1496	0.24	-	-	-		-	0.38	0.18	0.09	-	-
(*Z*,*E*)-α-Farnesene *	1496	0.27	-	-	-		-	0.15	-	-	-	0.42
Germacrene D *	1506	12.36	-	-	-	0.65	-	-	0.49	2.55	-	0.70
(*E*,*E*)-α-Farnesene *	1510	-	0.06	-	-	0.70	-	-	-	-	0.22	-
β-Selinene	1517	-	-	-	-		-	-	-	-	-	0.78
α-Muurolene	1527	-	-	-	2.17		-	-	-	-	-	1.35
α-Selinene	1529	-	-	-	-		-	-	-	-	-	0.50
(*Z*)-γ-Bisabolene	1535	-	-	-	-		-	-	-	-	-	0.69
β-Sesquiphellandrene	1542	-	-	-	-		-	-	-	-	-	0.69
δ-Cadinene	1542	1.36	-	-	-		-	-	-	0.26	-	0.42
α-Calacorene	1567	-	-	-	-		-	-	-	-	-	0.12
*Spiroacetals*												
1,6-Dioxaspiro[4.5] decane *	1057	-	-	0.41	0.64	0.01	0.06	-	-	-	-	0.01
(*E*)-Conophthorin *	1065	5.84	-	1.79	6.70	0.16	3.94	0.01	-	0.03	0.04	0.41
(*Z*)-Conophthorin *	1140	0.41	-	0.13	1.53		0.16	-	-	Tr	-	-
2-Methyl-1,7-dioxaspiro(5.5)undecane *	1152	-	-	-	-		-	-	-	-	-	0.14
7-Ethyl-1,6-dioxaspiro(4.5)decane *	1156	0.31	-	0.42	-		0.06	0.16	-	-	-	0.93
(*Z*)-7-Ethyl-1,6-dioxaspiro [4.5]decane *	1231	-	-	-	-		-	-	-	-	-	0.08
**Unidentified: (no. of compounds), % amount**		(11)	(15)	(9)	(1)	(4)	(0)	(8)	(9)	(10)	(3)	(9)
		23.61	5.09	9.90	1.19	11.08	-	4.34	11.27	3.40	1.79	3.28
**Total identified (%)**		76.39	94.91	90.1	98.81	88.92	100	95.66	88.73	96.6	98.37	96.72

* Identification confirmed with authentic standards; Tr—compounds found in relative amounts <0.01.
