# Supplementary material for: Differential Evolutionary History in Visual and Olfactory Floral Cues of the Bee-Pollinated Genus Campanula (Campanulaceae)
Source: Plants (Basel). 2021 Jul 2;10(7):1356. doi: 10.3390/plants10071356 (PMC8309401; doi:10.3390/plants10071356)
Supplement: Supplementary file 1 [file plants-10-01356-s001.zip › plants-1264805-supplementary/Table S1.pdf]

Table S1. Euclidean distances (in hexagon units) among flower colour loci of *Campanula* species (values ≤ 0.1 units are in bold). Distances to the hexagon centre (CEN) are also given. Abbreviations of species name are as follows: *C. glomerata* (GLO), *C. lactiflora* (LAC), *C. latifolia* (LAT), *C. medium* (MED), *C. moravica* (MOR), *C. patula* (PAT), *C. persicifolia* (PER), *C. punctata* (PUN), *C. rapunculoides* (RPC), *C. rapunculus* (RAP), *C. rotundifolia* (ROT), *C. scheuchzeri* (SCH), *C. thyrsoides* (THY) and *C. trachelium* (TRA).

	GLO	LAT	LAC	MED	MOR	PAT	PER	PUN	RPC	RAP	ROT	SCH	THY	TRA	CEN
GLO															0.25
LAT	**0.04**														0.22
LAC	0.33	0.29													0.20
MED	**0.10**	**0.08**	0.31												0.29
MOR	0.16	0.11	0.17	0.15											0.17
PAT	**0.09**	**0.06**	0.23	0.12	**0.07**										0.17
PER	0.21	0.17	0.14	0.17	**0.06**	0.13									0.21
PUN	0.23	0.19	**0.09**	0.22	**0.08**	0.14	**0.07**								0.15
RPC	**0.07**	**0.05**	0.30	**0.03**	0.13	**0.09**	0.17	0.21							0.26
RAP	**0.08**	**0.05**	0.26	0.13	0.10	**0.04**	0.16	0.17	0.10						0.17
ROT	0.23	0.19	0.12	0.20	**0.08**	0.15	**0.02**	**0.06**	0.19	0.18					0.21
SCH	**0.09**	**0.07**	0.28	**0.03**	0.12	**0.09**	0.14	0.19	**0.03**	0.12	0.17				0.27
THY	0.52	0.48	0.20	0.51	0.37	0.42	0.33	0.29	0.49	0.45	0.31	0.47			0.33
TRA	0.17	0.13	0.17	0.14	**0.04**	**0.09**	**0.04**	**0.09**	0.12	0.13	**0.06**	0.11	0.37		0.20
