# Supplementary material for: Differential Evolutionary History in Visual and Olfactory Floral Cues of the Bee-Pollinated Genus Campanula (Campanulaceae)
Source: Plants (Basel). 2021 Jul 2;10(7):1356. doi: 10.3390/plants10071356 (PMC8309401; doi:10.3390/plants10071356)
Supplement: Supplementary file 1 [file plants-10-01356-s001.zip › plants-1264805-supplementary/Table S3.pdf]

Table S3. Loading of wavelengths (300-700nm) in the PCs of the colour phylogenetic PCA (pPCA).

Wavelenghts (nm)	PC1	PC13
300	0.01602165	-0.04108138
310	0.03684538	-0.03774807
320	0.02680381	-0.03494278
330	0.03917892	-0.03044905
340	0.00849212	-0.03059024
350	-0.07573479	-0.038159
360	-0.17550491	-0.0641192
370	-0.22879529	-0.10333623
380	-0.23254095	-0.13340607
390	-0.23471548	-0.13927886
400	-0.25778107	-0.12214451
410	-0.26837313	-0.0948444
420	-0.22585075	-0.05030064
430	-0.14671571	0.00879697
440	-0.09936588	0.0555081
450	-0.07677404	0.09526556
460	-0.08561908	0.12893317
470	-0.09555745	0.15211905
480	-0.08980656	0.17295424
490	-0.06931516	0.19191662
500	-0.01076815	0.20793236
510	0.04393317	0.21837056
520	0.0880923	0.22632946
530	0.12091998	0.23106475
540	0.1392381	0.23160349
550	0.14285279	0.23011262
560	0.13054661	0.23005434
570	0.10140139	0.22526127
580	0.06370762	0.21797902
590	0.02964108	0.21191512
600	-0.00152968	0.21225044
610	-0.0706967	0.21582672
620	-0.14277262	0.21736388
630	-0.2082134	0.21178748
640	-0.25324035	0.19327452
650	-0.2695239	0.16228114
660	-0.25200185	0.13241359
670	-0.24994098	0.09694677
680	-0.23398637	0.07859761
690	-0.18939181	0.08695029
700	-0.1526773	0.0941343
