# Supplementary material for: Differential Evolutionary History in Visual and Olfactory Floral Cues of the Bee-Pollinated Genus Campanula (Campanulaceae)
Source: Plants (Basel). 2021 Jul 2;10(7):1356. doi: 10.3390/plants10071356 (PMC8309401; doi:10.3390/plants10071356)
Supplement: Supplementary file 1 [file plants-10-01356-s001.zip › plants-1264805-supplementary/Table S4.pdf]

Table S4. Scores of each *Campanula* species in the PCs of the colour phylogenetic PCA (pPCA).

Species	PC1	PC13
Campanula glomerata	-0.49809872	-0.09793997
Campanula lactiflora	-0.03454469	0.4489037
Campanula latifolia	-0.12967818	-0.17235813
Campanula medium	0.13030799	-0.44736924
Campanula moravica	0.1722332	-0.12551131
Campanula patula	-0.32396733	0.1138514
Campanula persicifolia	0.06197114	-0.11683345
Campanula punctata	0.04484866	0.14290352
Campanula rapunculoides	0.34257116	-0.49155058
Campanula rapunculus	-0.26456216	0.11840947
Campanula rotundifolia	0.26909938	-0.25334226
Campanula scheuchzeri	0.16937767	-0.38657881
Campanula thyrsoides	0.0739994	1.42260855
Campanula trachelium	-0.01355753	-0.15519292
