# Supplementary material for: Differential Evolutionary History in Visual and Olfactory Floral Cues of the Bee-Pollinated Genus Campanula (Campanulaceae)
Source: Plants (Basel). 2021 Jul 2;10(7):1356. doi: 10.3390/plants10071356 (PMC8309401; doi:10.3390/plants10071356)
Supplement: Supplementary file 1 [file plants-10-01356-s001.zip › plants-1264805-supplementary/Table S5.pdf]

Table S5. Loading of compounds in the PCs of the scent phylogenetic PCA (pPCA).

Compounds	PC1	PC10
(*E*)-2-Hexenal	-0.0693656	-0.08087125
(*E*)-2-Hexenol	-0.07978117	-0.09301444
(*Z*)-3-Hexenol	-0.10202736	-0.1189506
(*E*)-Epoxyocimene	0.04012247	-0.05935527
(*E*)-Linalol oxide (furanoid)	-0.10980609	-0.07411437
(Z)-Linalol oxide (furanoid)	-0.03928024	-0.04579564
(*Z*)-Ocimenone	0.00171716	-0.00474678
(*Z.E*)-α-Farnesene	-0.0591046	-0.0405051
1.6-Dioxaspiro [4.5] decane	-0.0556249	0.08595418
1-Nitro-2-phenyl ethane	-0.05679561	0.06817507
1-Phenylethanol	0.07333081	-0.01893703
2-Methyl-1.7-dioxaspiro(5.5)undecane	-0.01035379	-0.04752302
2-Nonanone	-0.01803162	-0.08276361
2-Phenylethanol	0.15531232	-0.05567701
2-Phenylethyl acetate	0.08797553	-0.00692894
4-Ethylguaiacol	0.05001585	-0.01291615
7-Ethyl-1.6-dioxaspiro(4.5)decane	-0.06702593	-0.0099324
α- Calacorene	-0.00993617	-0.0456062
α-Cubebene	-0.05227661	0.05816533
Allo-aromadendrene	-0.04603896	0.11216819
Allo-ocimene	0.03344949	-0.07544967
α-Longipinene	0.04543027	-0.04300755
α-Caryophyllene	0.09055542	0.01830667
α-Ylangene	0.19210808	0.19210182
Amorpha-4.11-diene	-0.01607924	-0.07380235
α-Phellandrene	-0.05627377	-0.06560788
α-Pinene	0.08167406	0.00455027
Ar-Curcumene	0.04724599	-0.05990512
Artemisiatriene	-0.0285928	-0.03333547
α-Selinene	-0.01431237	-0.06569257
α-Terpinene	-0.14966631	0.03402521
α-Terpineol	0.10246583	-0.09875496
β-Bourbonene	0.10289101	0.148995
β-Citronellol	-0.06706116	-0.07818457
B-Cubenene	-0.04221558	0.02060455
B-Elemene	-0.0202326	-0.09286594
Phenylacetaldehyde	0.10637166	0.06044502
Benzeneacetonitrile	-0.02158913	0.09953997
Benzyl acetate	0.15021665	0.02336106
Benzyl alcohol	0.17131451	-0.08090526
β-Sesquiphellandrene	-0.0155423	-0.07133785
β-Phellandrene	-0.05951797	-0.0693902
β-Pinene	0.05792306	0.00322704

β-Selinene	-0.01600848	-0.07347759
β-Cedrene	0.1708763	0.06340781
β-gurjunene	0.0545946	0.02477274
(*Z*)-3-Hexenyl acetate	0.16580425	-0.29637022
(*Z*)-γ-Bisabolene	-0.01550796	-0.07118026
(*Z*)-ß-Ocimene	0.01560755	-0.21977801
Citronellal	-0.04011469	-0.0467685
δ-3-Carene	-0.02539568	0.03028099
δ-Cadinene	0.03593022	-0.0681997
Dill ether	-0.04788272	-0.06441345
(*E.E*)-α-Farnesene	0.01412175	-0.05815205
(*E*)-β-Caryophyllene	0.18111922	0.03854019
(*E*)-β-Farnesene	0.02729727	-0.04953777
Geranial	-0.09156277	-0.10675025
(*E*)-Conophthorin	-0.13479715	0.08138447
Geraniol	-0.15526262	-0.18101597
(*E*)-Isocitral	-0.04689745	-0.05467631
Eucalyptol	-0.26060593	0.16087565
γ -Muurolene	0.04712857	0.03779021
γ -Terpinene	-0.04938281	-0.05757392
Geranyl acetone	-0.11549011	0.05301376
Germacrene D	0.20071244	-0.04710273
Guaiacol	-0.01842531	0.01822362
Hexyl acetate	0.09406777	-0.10272058
Indole	0.0356259	0.02483284
4-Oxoisophorone	-0.03118064	0.14396659
Lavandulol	-0.07435631	0.00061655
Lavandulyl acetate	-0.03046338	0.06334293
Limonene	-0.09964932	-0.11617811
Linalool	-0.07353547	-0.23753917
Linalool oxide (pyranoid)	-0.11400614	-0.002368
Longifolene	-0.00902698	-0.04143308
Methyl geranate	-0.03368174	-0.03926852
Methyl salicilate	0.17435271	-0.13007537
Myrcene	0.07418689	-0.06922133
Neo-allo-ocimene	0.01642494	-0.03901334
Nerol oxide	-0.04091546	-0.04770209
Hexanol	-0.07505527	-0.08750466
o-Anisaldehyde	-0.01207214	0.0093425
o -Aminoacetophenone	0.0107055	0.03106285
p-Anysaldehyde	-0.06395019	0.0316811
p-Cymene	-0.01381879	-0.0634271
p-Mentha-1.5-dien-8-ol	0.00191857	-0.00530353
p-Methylanisole	-0.01172172	0.10368717
Prezizaene	0.1910022	-0.04316733
Sabinene	-0.01039333	0.01112665

Sesquiterpene 1	-0.01234028	0.00955001
Sesquiterpene 10	0.06252413	0.00348338
Sesquiterpene 11	-0.0620279	-0.03161606
Sesquiterpene 12	-0.00813133	0.02931106
Sesquiterpene 13	0.15536563	0.00576658
Sesquiterpene 14	0.1417541	0.0322957
Sesquiterpene 15	-0.0091263	0.01434156
Sesquiterpene 16	0.09101384	0.04129826
Sesquiterpene 17	0.1062657	-0.03626645
Sesquiterpene 18	0.18079055	0.03882454
Sesquiterpene 19	0.14329261	0.11814511
Sesquiterpene 2	0.05919195	-0.01098428
Sesquiterpene 20	0.09646248	-0.02491057
Sesquiterpene 21	0.15426822	0.08908532
Sesquiterpene 22	0.13830488	0.02568807
Sesquiterpene 3	0.00029971	0.06687707
Sesquiterpene 4	-0.03686118	0.01807474
Sesquiterpene 5	0.00025954	0.05791256
Sesquiterpene 6	-0.00546438	-0.04129514
Sesquiterpene 7	-0.06883642	0.02913674
Sesquiterpene 8	-0.02282651	0.08903864
Sesquiterpene 9	0.04695501	-0.0270496
Terpinolene	-0.03981937	-0.11058679
(*E*)-α-Bergamotene	-0.02045117	-0.09386916
(*E*)-ß-Ocimene	0.159355	-0.37219657
Tridecane	-0.02495064	-0.11452135
Unknown 1	0.00260978	-0.00721425
Unknown 10	-0.04529871	-0.05281239
Unknown 11	-0.00885463	0.0068525
Unknown 12	-0.04954776	0.02592347
Unknown 13	-0.08887038	-0.0187659
Unknown 14	-0.07451232	-0.08687164
Unknown 15	-0.05848675	-0.06818792
Unknown 16	0.00450562	-0.01245498
Unknown 17	0.00243234	-0.00672376
Unknown 18	0.00215562	-0.00595883
Unknown 19	0.00215591	-0.00595962
Unknown 2	0.00227936	-0.00630088
Unknown 20	0.00196753	-0.00543887
Unknown 21	-0.05069783	0.02755569
Unknown 22	-0.00991323	-0.05574573
Unknown 23	-0.08547711	0.08312947
Unknown 24	0.00203798	-0.00563361
Unknown 25	0.00216718	-0.00599078
Unknown 26	0.06195385	-0.01599903
Unknown 3	-0.00800364	-0.03673603

Unknown 4	0.00028884	0.06445159
Unknown 5	0.00043615	0.09732159
Unknown 6	0.00247619	-0.00684498
Unknown 7	0.00196361	-0.00542805
Unknown 8	0.00233631	-0.0064583
Unknown 9	0.05092649	-0.00519274
Verbenone	-0.00792953	-0.03639589
Ylanga-2.4(15)-diene	0.00021852	0.04876039
(*Z*)-7-Ethyl-1.6-dioxaspiro(4.5)decane	-0.00904203	-0.04150216
Neral	-0.08729771	-0.10177775
(*Z*)-Conophthorin	-0.07747901	0.1136441
Nerol	-0.15638515	-0.18232469
(*Z*)-Isocitral	-0.03919337	-0.04569435
α-Copaene	-0.05452854	0.1459617
α-Muurolene	-0.10699342	0.00786171
