# Supplementary material for: Differential Evolutionary History in Visual and Olfactory Floral Cues of the Bee-Pollinated Genus Campanula (Campanulaceae)
Source: Plants (Basel). 2021 Jul 2;10(7):1356. doi: 10.3390/plants10071356 (PMC8309401; doi:10.3390/plants10071356)
Supplement: Supplementary file 1 [file plants-10-01356-s001.zip › plants-1264805-supplementary/Table S6.pdf]

Table S6. Scores of *Campanula* species in the PCs of the scent Phylogenetic PCA (pPCA).

Species	PC1	PC10
Campanula glomerata	-0.11390973	1.18967664
Campanula lactiflora	0.01667072	-1.76237417
Campanula latifolia	-0.31426454	2.72387947
Campanula medium	-2.67116754	2.44111151
Campanula persicifolia	1.50436125	-0.55309364
Campanula punctata	-0.28099431	-0.22567728
Campanula rapunculoides	-1.10362469	1.56383615
Campanula rapunculus	2.77874624	1.27164327
Campanula rotundifolia	2.48403147	-0.68151923
Campanula thyrsoides	-2.7362542	-3.28800931
Campanula trachelium	0.43640534	-2.6794734
