# Supplementary figures and images for: Differential Evolutionary History in Visual and Olfactory Floral Cues of the Bee-Pollinated Genus Campanula (Campanulaceae)
Source: Plants (Basel). 2021 Jul 2;10(7):1356. doi: 10.3390/plants10071356 (PMC8309401; doi:10.3390/plants10071356)

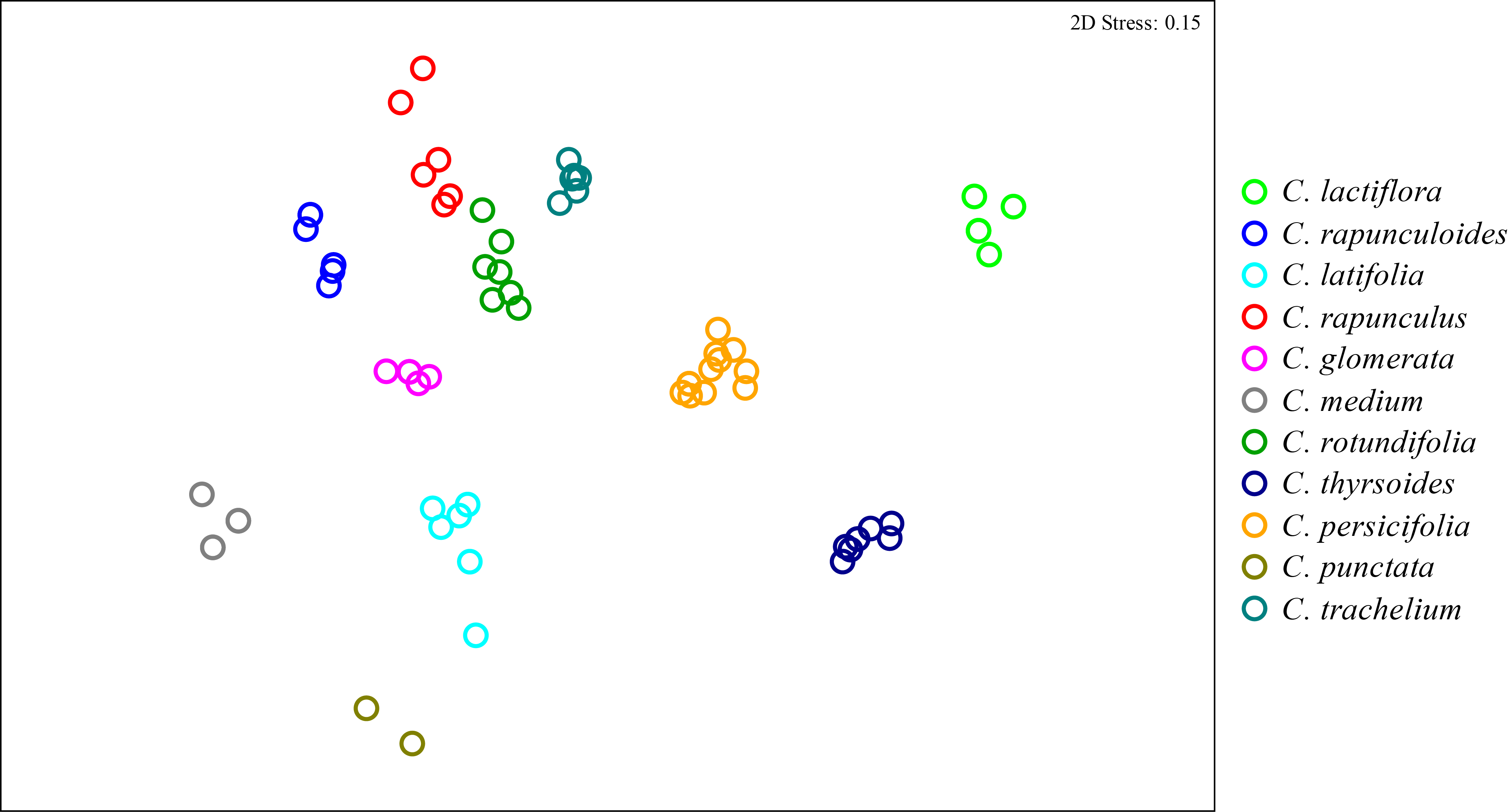

Supplement: Supplementary file 1 [file plants-10-01356-s001.zip › plants-1264805-supplementary/Fig. S1 - Milet-Pinheiro et al. 2021 Plants_MDPI.png]
